# Gallbladder Cancer Predisposition: A Multigenic Approach to DNA-Repair, Apoptotic and Inflammatory Pathway Genes

**DOI:** 10.1371/journal.pone.0016449

**Published:** 2011-01-21

**Authors:** Kshitij Srivastava, Anvesha Srivastava, Ashok Kumar, Balraj Mittal

**Affiliations:** 1 Department of Genetics, Sanjay Gandhi Post Graduate Institute of Medical Sciences, Lucknow, India; 2 Department of Surgical Gastroenterology, Sanjay Gandhi Post Graduate Institute of Medical Sciences, Lucknow, India; Universidade de São Paulo, Brazil

## Abstract

Gallbladder cancer (GBC) is a multifactorial disease with complex interplay between multiple genetic variants. We performed Classification and Regression Tree Analysis (CART) and Grade of Membership (GoM) analysis to identify combinations of alleles among the DNA repair, inflammatory and apoptotic pathway genetic variants in modifying the risk for GBC. We analyzed 16 polymorphisms in 8 genes involved in DNA repair, apoptotic and inflammatory pathways to find out combinations of genetic variants contributing to GBC risk. The genes included in the study were *XRCC1*, *OGG1*, *ERCC2*, *MSH2*, *CASP8*, *TLR2*, *TLR4* and *PTGS2*. Single locus analysis by logistic regression showed association of *MSH2* IVS1+9G>C (rs2303426), *ERCC2* Asp312Asn (rs1799793), *OGG1* Ser326Cys (rs1052133), *OGG1* IVS4-15C>G (rs2072668), *CASP8* -652 6N ins/del (rs3834129), *PTGS*2 -1195G>A (rs689466), *PTGS2* -765G>C (rs20417), *TLR4* Ex4+936C>T (rs4986791) and *TLR2* –196 to –174del polymorphisms with GBC risk. The CART analysis revealed *OGG1* Ser326Cys, and *OGG1* IVS4-15C>G polymorphisms as the best polymorphic signature for discriminating between cases and controls. In the GoM analysis, the data was categorized into six sets representing risk for GBC with respect to the investigated polymorphisms. Sets I, II and III described low intrinsic risk (controls) characterized by multiple protective alleles while sets IV, V and VI represented high intrinsic risk groups (GBC cases) characterized by the presence of multiple risk alleles. The CART and GoM analyses also showed the importance of *PTGS2 -*1195G>A polymorphism in susceptibility to GBC risk. In conclusion, the present multigenic approach can be used to define individual risk profiles for gallbladder cancer in North Indian population.

## Introduction

Carcinoma of the gallbladder (GBC) is an aggressive malignancy and the most common biliary tract tumor in the world with highest incidence and mortality rates in Northern India (21.5/100,000) [1], [2]. Apart from gallstones being the major risk factor, the exact etiology of GBC is poorly understood [3]. Cancer being a multifactorial disease, multiple genetic variants along with the environmental and dietary factors may interact to cause the disease or act as risk modifiers. Identification of these risk sets of genetic variants causative to the disease will facilitate in determining individuals at risk for GBC.

Previously, we have studied the role of individual genetic variants with GBC susceptibility in North Indian population [4], [5], [6]. Due to conflicting results obtained in case-control association studies of complex diseases such as cancer, the current focus is aimed on searching for gene-gene interactions as a key contributory factor in the disease outcome. But the analysis of such interactions in case-control studies is weighed down by one of the major problems, namely, the curse of dimensionality. Recently, Multifactor-Dimensionality Reduction (MDR) approach [7] and tree-based techniques, classification and regression trees (CART) and random forest (RF), have been used to detect interactions in large-scale association studies [8]. The strength of these methodologies is their ability to identify association in cases of small sample sizes and low penetrance of candidate single nucleotide polymorphisms (SNPs). Therefore, we have extended our previous work by jointly investigating 16 SNP genotypes in 8 genes belonging to DNA repair pathway [*ERCC2* Asp312Asn (Ex10-16G>A; rs1799793) and Lys751Gln (Ex23+61A>C; rs13181); *MSH2* (IVS1+9G>C; rs2303426) and (-118T>C; rs2303425); *OGG1* Ser326Cys (Ex6-315C>G; rs1052133) and (IVS4-15C>G; rs2072668); *XRCC1* Arg194Trp (Ex6-22C>T; rs1799782) and Arg399Gln (Ex10-4A>G; rs25487)], apoptotic pathway [*CASP8* -652 6N ins/del (rs3834129), Asp302His (Ex13+51G>C; rs1045485) and (IVS12-19G>A; rs3769818)] and inflammatory pathway [*PTGS*2 (-1195G>A; rs689466), (-765G>C; rs20417) and (Ex10+837T>C or +8473; rs5275); *TLR2* –196 to –174del (*TLR2* Δ22); and *TLR4* Thr399Ile (Ex4+936C>T; rs4986791)], avoiding the problem of dimensionality and multiple comparisons. These polymorphisms have been reported to alter the risk for developing various malignancies [9], [10], [11], [12], [13], [14].

## Materials and Methods

### Ethics Statement

The institutional ethical committee of Sanjay Gandhi Post Graduate Institute of Medical Sciences (SGPGIMS) approved the study protocol, and all participants provided written informed consent for the study.

### Study Population

A total of 460 subjects, including 230 GBC patients and 230 control subjects were enrolled in this study. The GBC patients were consecutively diagnosed between June 2005 and September 2009. Gallbladder cancer diagnosis was confirmed for all cases by fine needle aspirated cell cytology (FNAC) and histopathology. Staging of cancer was documented according to the AJCC/UICC staging [15]. The inclusion criteria for controls were absence of prior history of cancer, precancerous lesions and gallstones proven by ultrasonography and were frequency-matched to cancer cases on age, gender and ethnicity. To test the possibility for population stratification, genomic control method was used as described by Devlin et al [16]. Majority of the female patients were housewives and the male patients were not engaged in any hazardous occupations.

### Genotyping

Genomic DNA was isolated from peripheral blood leukocytes. The polymorphisms were genotyped using the PCR or PCR-restriction fragment length polymorphism method. The details of genotyping for studied polymorphisms are shown in Table S1. As a negative control, PCR mix without DNA sample was used to ensure contamination free PCR product. Samples that failed to genotype were scored as missing. Genotyping was performed without knowledge of the case or control status.

### Statistical Analysis

#### Single Locus Analysis

The sample size was calculated considering the minor allele frequency (MAF) of the studied polymorphisms in Caucasian population. The sample size of 230 cases and 230 controls was adequate to give us a power of 80% (Inheritance mode =  log-additive, Genetic effect = 2, Type-I error rate = 0.05). Chi-square analysis or two-sided Fisher's exact test was used to compare the differences in demographic variables and genotype distributions of the polymorphisms between cases and controls. Observed genotype frequencies for all the polymorphisms in controls were examined for deviation from Hardy–Weinberg equilibrium (HWE) using a goodness-of-fit χ^2^-test with one degree of freedom. Unconditional univariate and multivariate logistic regression analysis was used to estimate odds ratio (OR) and 95% confidence interval (CI) adjusted for age and gender to estimate the risk of gallbladder cancer with the polymorphisms. Risk estimates were also calculated for a codominant genetic model using the most common homozygous genotype as reference. Tests of linear trend using an ordinal variable for the number of copies of the variant allele (0, 1 or 2) were conducted to assess potential dose–response effects of genetic variants on gallbladder cancer risk [17]. Standard adjustments for multiple testing, such as Bonferroni correction, are too conservative as they assume that tests are independent, which is usually not the case when multiple tests are applied on the same data set. We, therefore applied the false-positive report probability (FPRP) statistical tool to evaluate noteworthiness of the associations by using the method as described by Wacholder et al [18].

To further support the results of logistic regression, we used genomic control method by Devlin et al [16]. The software uses a Bayesian outlier test to determine which markers exhibit significant linkage disequilibrium with the disorder minimizing the false positive associations. Also it allows for violations in the usual model assumption i.e. independence of observations because in case-control studies affected individuals are more likely to be related than are controls because they share a genetic disorder and ideally a common genetic basis for the disorder. This is known as “cryptic relatedness” which almost always produces false positives even after Bonferroni correction. The software performs these analyses using Markov chain Monte Carlo (MCMC) algorithms.

#### Classification and Regression Tree Analysis (CART)

The non-parametric classification and regression tree analysis was used along with the logistic regression for higher-order gene-gene interactions using the CART Software (version 6.0, Salford Systems) [19], [20]. CART is a binary recursive-partitioning method that produces a decision tree to identify subgroups of subjects at higher risk. Specifically, the recursive-partitioning algorithm in CART software starts at the first node (with the entire data set) and uses a statistical hypothesis-testing method—formal inference based recursive modelling—to determine the first locally optimal split and each subsequent split of the data set, with multiplicity-adjusted *P*-values to control tree growth. This process continues until the terminal nodes have no subsequent statistically significant splits or the terminal nodes reach a pre-specified minimum size. The data were divided randomly into a learning set (90% of the data) and a testing set (10% of the data). The learning set was used to construct the tree model, and the testing set was used to internally validate the resulting tree model. Subgroups of individuals with differential risk patterns were then identified in the different order of nodes of the tree structure, indicating the presence of gene–gene and gene–environment interactions. Logistic regression was used to calculate the OR and 95% CI in each terminal node of the tree, adjusting for age and gender. *P*<0.05 was considered as the threshold of significance in this study. All statistical analyses were two-sided.

#### Grade of Membership Analysis (GoM)

To investigate all the genetic factors simultaneously without multiple comparisons, the Grade of Membership (GoM) analysis was used [21], [22]. Information on disease status and genetic variants were used as internal variables to define the pure types while sex and age were external variables. The approach taken is termed as latent classification method that is considered to be ‘fuzzy’ in that each individual can partially belong to more than one group, here, genetic risk sets. The GoM analysis framework thus makes very few distributional assumptions. The GoM models were constructed as described previously [23].

## Results

### Population characteristics

Baseline characteristics of GBC patients and their age and gender matched controls are presented in Table 1. Of the 230 GBC cases and controls, the mean age was 53.05±6.40 years (range, 37–72 years) and 54.12±8.81 (range, 35–79 years), respectively. The mean age and gender distributions were not significantly different among cases and controls, suggesting that the frequency matching was adequate. Genomic control method ruled out the possibility of population stratification in our study. Most of the GBC patients were in advanced stages of cancer (stage III and stage IV). Of the 230 GBC cases, 12 (5.3%) had stage II adenocarcinoma, 76 (33.0%) stage III and 142 (61.7%) stage IV. All cancer patients were incident cases and none of the controls had family history of cancer.

**Table 1:** Characteristic profile of controls and GBC patients.

Characteristic	Healthy controls (N = 230)	GBC case patients (N = 230)
Gender, *n* (%)		
Male	78(33.9)	79(34.3)
Female	152(66.1)	151(65.7)
Age, mean (SD*)	54.12±8.81	53.05±6.40
Stages, *n* (%)		
0, I		None
II		12(5.3)
III		76(33.0)
IV		142(61.7)
Gallstone present, n (%)	None	117(51.0)

*SD, standard deviation.

### Single Locus Analysis

Table 2 shows the GBC risk related to the studied polymorphisms.

**Table 2:** Single locus analysis of SNPs investigated.

Gene	Polymorphism	MAF_controls_	MAF_cases_	*P*_trend_	FPRP	OR_het_ a	OR_hom_ a	OR_dom_ a	Prob^b^
***ERCC2***	Asp312Asn (rs1799793)	0.30	0.34	0.041	0.922	0.91 (0.60–1.42)	**2.12 (1.12–4.01)**	1.14 (0.72–1.62)	0.445
***ERCC2***	Lys751Gln (rs13181)	0.31	0.37	0.163	0.673	1.45 (0.92–2.01)	1.54 (0.88–2.67)	1.43 (0.91–2.10)	0.453
***MSH2***	-118T>C (rs2303425)	0.17	0.17	0.812	0.930	0.86 (0.67–1.43)	1.39 (0.57–4.05)	0.88 (0.67–1.58)	0.367
***MSH2***	IVS1+9G>C (rs2303426)	0.48	0.56	0.052	0.849	1.15 (0.73–1.79)	**1.83 (1.13–3.14)**	1.34 (0.89–2.18)	0.492
***OGG1***	Ser326Cys (rs1052133)	0.23	0.29	0.070	0.044	1.32 (0.91–1.90)	**2.48 (1.13–5.42)**	**1.78 (1.22–2.63)**	0.502
***OGG1***	IVS4-15C>G (rs2072668)	0.33	0.42	0.027	0.338	1.52 (1.01–2.29)	**2.01 (1.22–3.58)**	**1.63 (1.14–2.43)**	0.531
***XRCC1***	Arg194Trp (rs1799782)	0.11	0.12	0.618	0.933	0.94 (0.58–1.54)	1.68 (0.27–10.25)	0.92 (0.59–1.57)	0.390
***XRCC1***	Arg399Gln (rs25487)	0.44	0.32	<0.001	0.128	**0.57 (0.37–0.87)**	**0.44 (0.22–0.76)**	**0.55 (0.35–0.86)**	0.598
***PTGS2***	-1195G>A (rs689466)	0.13	0.23	<0.001	0.023	**1.99 (1.17–3.40)**	**7.04 (2.23–22.19)**	**2.54 (1.55–4.16)**	0.599
***PTGS2***	-765G>C (rs20417)	0.08	0.18	<0.001	0.056	**1.91 (1.23–2.97)**	2.74 (0.95–7.90)	**1.99 (1.31–3.05)**	0.612
***PTGS2***	Ex10+837T>C (rs5275)	0.39	0.42	0.334	0.692	1.40 (0.91–2.16)	1.34 (0.74–2.45)	1.39 (0.92–2.09)	0.408
***TLR2***	–196 to –74del	0.19	0.23	0.091	0.364	**1.51 (1.02–2.24)**	2.14 (0.56–8.11)	**1.54 (1.05–2.26)**	0.465
***TLR4***	Thr399Ile (rs4986791)	0.06	0.09	0.021	0.445	1.74 (0.96–3.16)	7.57 (0.83–69.28)	**1.96 (1.11–3.49)**	0.504
***CASP8***	-652 6N ins/del (rs3834129)	0.29	0.20	0.005	0.154	**0.66 (0.44–0.98)**	**0.42 (0.20–0.89)**	**0.61 (0.42–0.88)**	0.539
***CASP8***	IVS12-19G>A (rs3769818)	0.14	0.16	0.375	0.803	1.33 (0.87–2.02)	0.83 (0.18–3.89)	1.30 (0.86–1.96)	0.414
***CASP8***	Asp302His (rs1045485)	0.04	0.05	0.421	0.894	1.36 (0.70–2.64)	0.95 (0.06–15.5)	1.33 (0.69–2.56)	0.402

a Adjusted for age and gender in logistic regression model, Significant values are denoted as bold.

FPRP, false-positive report probability based on OR_dom_ (assuming prior probability of 0.05 and power to detect an OR of 2.0 or 0.5).

OR_het_, odds ratio of heterozygote vs. common homozygote genotypes, OR_hom_, odds ratio of rare homozygote vs. common homozygote genotypes, OR_dom_, odds ratio of heterozygote+variant homozygote vs. common homozygote genotypes.

b Probability of association in genomic control method (Minimum = 0.5).

#### Association of ERCC2, MSH2, XRCC1 and OGG1 polymorphisms with GBC

On comparing the genotype frequency distribution in GBC patients with that of controls, the homozygous variant genotypes of *MSH2* IVS1+9G>C, *ERCC2* Asp312Asn and *OGG1* Ser326Cys polymorphisms showed statistically significant increased risk for developing GBC (OR = 1.83; OR = 2.12; OR = 2.48, respectively). The risk due to variant-containing genotypes (CG+GG) of *OGG1* Ser326Cys was also significant (OR = 1.78) when compared with homozygous wild-type CC genotype. The *OGG1* IVS4-15C>G intronic polymorphism was also significantly associated with the risk for GBC following a dominant mode of inheritance (OR = 1.63) (Table 2).

In case of *XRCC1* Arg399Gln polymorphism, frequencies of heterozygote and variant homozygote were significantly different among in controls as compared to GBC patients (χ^2^ = 13.84; *P* = 0.001; df, 2). These frequency differences were statistically significant (*P* = 0.039; *P* = 0.003 respectively) and conferred low risk for GBC (OR = 0.57; OR = 0.44 respectively) (Table 2). The protective effect was also significant when variant containing genotypes (GA+AA) were compared with homozygous wild-type genotype (*P* = 0.006; OR = 0.55) (Table 2).

For the other SNPs in DNA repair genes (*ERCC2* Lys751Gln, *MSH2* -118T>C and *XRCC1* Arg194Trp), no statistically significant associations were observed in the present study (Table 2).

#### Association of CASP8 polymorphisms with GBC

Frequencies of the *CASP8* -652 6N common homozygote, heterozygote and variant homozygote genotypes were significantly different among GBC patients and controls (χ^2^ = 7.79; *P* = 0.020; df, 2). In the present study, we found that both heterozygote and variant homozygote genotypes were associated with a statistically significant decreased risk of GBC (OR = 0.66; OR = 0.42, respectively (Table 2) with the ‘del’ allele being associated with the decreased risk of gallbladder cancer in a dose-dependent manner (*P*_trend_  = 0.003). Furthermore, a significant decreased risk of GBC was found with the *CASP8* -652 (ins/del + del/del genotypes) compared with the -652 6N ins/ins genotype, suggesting a dominant protective effect of this polymorphism on GBC (OR = 0.61; 95% CI = 0.42-0.88; *P*_trend_ = 0.005). No significant association was found between IVS12-19G>A and Ex13+51G>C polymorphisms and overall GBC risk.

#### Association of PTGS2 polymorphisms with GBC

On comparing the genotype frequency distribution in GBC patients with that of controls, the frequency of *PTGS2* -1195 heterozygote and variant homozygote genotypes were associated with significant increased risk (OR = 1.99; OR = 7.04; respectively) for GBC (Table 2). The trend test was also significant (*P*_trend_<0.001). The risk due to variant-containing genotypes (GA+AA) was significant (*P*<0.001; OR = 2.54) when compared with homozygous wild-type GG genotype. The *PTGS2* -765 GC genotype was also associated with significant increased risk (OR = 1.91; 95%CI = 1.23-2.97) for GBC (Table 2). The trend test was also significant (*P*_trend_<0.001). However, none of the genotypes of *PTGS2* +8473T>C polymorphisms were significantly associated with susceptibility of gallbladder cancer (Table 2).

#### Association between TLR polymorphisms and GBC risk

Table 3 shows the GBC risk related to the *TLR2* (Δ22) and *TLR4* (Ex4+936C>T) polymorphisms. In the present study, we found that both heterozygote and variant homozygote genotypes of both the *TLR* polymorphisms were associated with increased risk of GBC (OR = 1.51; OR = 2.14; *P*_trend_ = 0.091; Table 2). Furthermore, a significant increased risk of GBC was found with the *TLR2* (Δ22) (ins/del + del/del genotypes) as compared with Δ22 ins/ins genotype, suggesting a dominant effect model involved in the risk of this polymorphism on GBC (OR = 1.54; 95% CI = 1.05-2.26; *P*_trend_ = 0.117).

**Table 3:** Risk estimates of CART terminal nodes.

Node	Genotype of Individuals in Each Node	Case	Control	Case Rate^a^ (%)	Adjusted OR^b^ (95% CI)
Node 1	*OGG*-326(W) + *PTGS*-765(W) + *TLR*-399(M) + *CASP*-302(M)	1	9	10.0	Reference
Node 2	*OGG*-326(W) + *PTGS*-765(W) + *TLR*-399(W) + *CASP*-302(W)	55	109	33.5	3.21 (0.37–27.41)
Node 3	*OGG*-326(M) + *PTGS*-1195(W) + *PTGS*-8473(W)	18	31	36.7	3.95 (0.44–35.79)
Node 4	*OGG*-326(M) + *PTGS*-1195(W) + *PTGS*-8473(M) + *XRCC*-399(M)	25	32	43.9	5.30 (0.59–47.29)
Node 5	*OGG*-326(W) + *PTGS*-765(M)	38	19	66.7	13.78 (1.54–123.46)
Node 6	*OGG*-326(M) + *PTGS*-1195(M)	43	19	69.4	13.84 (1.55–123.72)
Node 7	*OGG*-326(M) + *PTGS*-1195(W) + *PTGS*-8473(M) + *XRCC*-399(W)	27	11	71.1	18.47 (1.97–173.12)
Node 8	*OGG*-326(W) + *PTGS*-765(W) + *TLR*-399(M) + *CASP*-302(W)	18	0	100.0	-
Node 9	*OGG*-326(W) + *PTGS*-765(W) + *TLR*-399(W) + *CASP*-302(M)	5	0	100.0	-

a Case rate is the percentage of cancer patients among all individuals in each node [case/(case + control) x 100].

b Adjusted for age and gender.

The *TLR4* Ex4+936C>T polymorphism was also found to be significantly associated with the overall GBC risk under a dominant mode of inheritance (OR = 1.96; 95% CI = 1.11-2.26; *P*_trend_ = 0.021).

### CART Analysis

**Figure 1:**
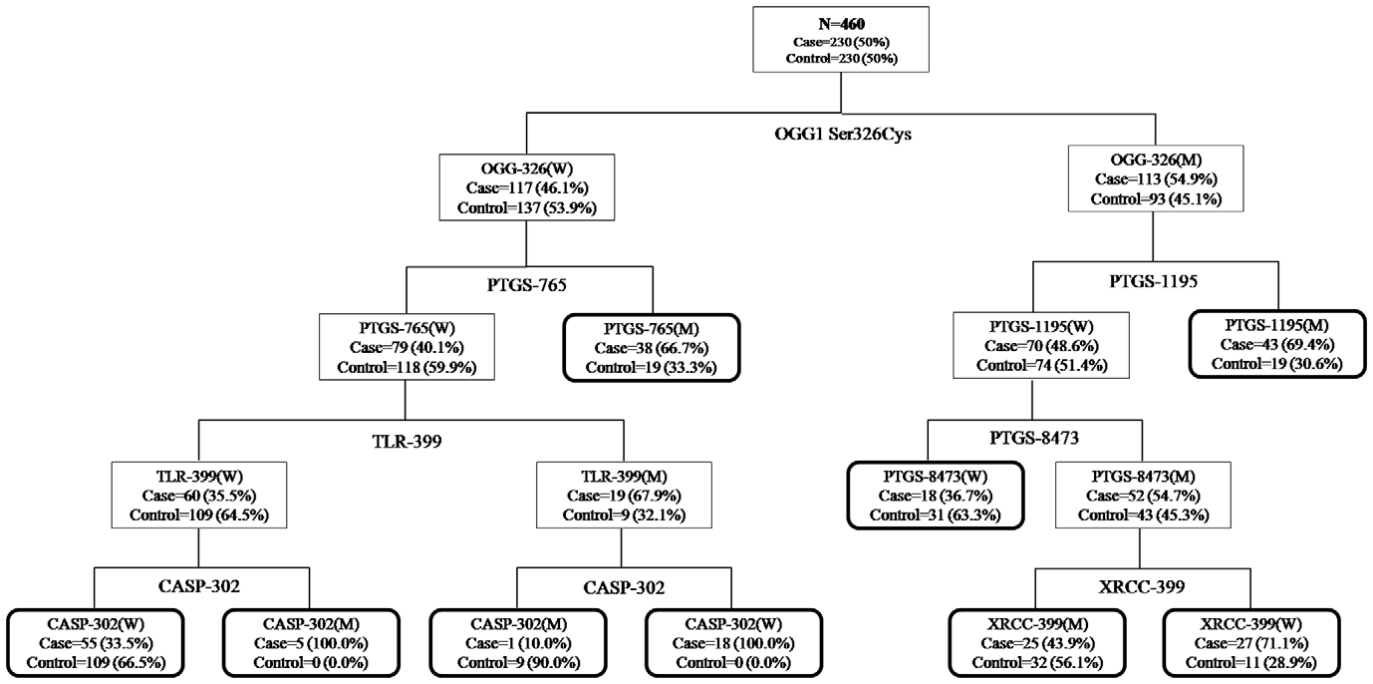
CART analysis of genetic variants in DNA repair, Apoptotic and Inflammatory pathway genes in modulating GBC risk. W = common homozygote genotypes, M = heterozygote+variant homozygote genotypes.

Figure 1 depicts the tree structure generated using the CART, which included all investigated genetic variants of the DNA repair, apoptotic and inflammatory pathway. The final tree structure contained six terminal nodes as defined by single-nucleotide polymorphisms of the DNA repair, apoptotic and inflammatory pathway genes. The *OGG1* Ser326Cys genotype was singled out in the first splitting node, separating individuals with the wild type–containing genotypes (low risk) from subjects with the homozygous variant genotype (high risk). Individuals with the variant genotypes of *CASP8* Asp302His, *TLR4* Thr399Ile and wild type genotypes of *OGG1* Ser326Cys, *PTGS2* -765G>C exhibited the lowest GBC risk with a 10% case rate (Fig. 1). Table 3 summarizes the risk associated with all the terminal subgroups compared with the subgroup with the least case percentage (Node 1). Table 4 shows the OR estimates generated for the three different risk groups determined on the basis of the case ratio of each CART terminal node. Compared with the low-risk group combining terminal nodes with a case ratio less than 40%, the medium-risk (case ratio between 40% and 70%) and high-risk groups (case ratio more than 70%) were both associated with a significantly increased GBC risk with ORs of 3.08 (95% CI = 1.98-4.77) and 10.04 (95% CI = 4.76-21.19), respectively (*P*_trend_<0.001).

**Table 4:** Combined analysis of the effects of genetic variants in the DNA repair, apoptotic and inflammatory pathways on GBC risk based on the results of the CART analysis.

Risk group^a^	Control/Case (N)	OR (95% CI)^b^	*P*-value
Low-risk	109/74	1.0	-
Medium-risk	70/106	3.08(1.98–4.77)	<0.001
High-risk	11/50	10.04(4.76–21.19)	<0.001
*P*_trend_			<0.001

a Low risk: case ratio<40, medium risk: 40≤case ratio<70, high risk: case ratio≥70.

b Adjusted for age and gender.

### GoM Analysis

Six groups categorized the data distinguishing high from how intrinsic risk for GBC with respect to the examined variants (Table S2): Groups I, II, and III were the control subjects (low-risk); groups IV, V and VI were the GBC patients (high-risk) (Table S2).

#### Low-risk groups

The low intrinsic risk (without disease) was characterized by multiple protective genotypes (i.e., groups I, II and III) (Table S2). These low-risk sets had the most common genotypes for the studied variables over-represented, denoting protection. Some of the variant alleles were also over-represented in the low-risk sets implying that many control subjects carried some risk for GBC. Group I had the age at enrolment of 56–60 years. The group included around 89% female subjects. They were 100% carriers of the wild allele for the *PTGS2* (-1195G>A), with a QRF of 1.54. A QRF of one is neutral. Group II were 100% carriers of the *OGG1* (IVS4-15C>G) ‘C’ allele and *OGG1* Ser326Cys ‘Cys’ allele with a QRF of 1.29 and 1.30, respectively. 50% of this group also carried *MSH2* (IVS1+9G>C) variant ‘C’ allele. Group III were heterozygous wild for the *TLR4* Thr399Ile and TLR2 –196 to –174del variant allele with a QRF of 1.55 and 1.44 respectively. This group was also homozygous for *PTGS2* -765G>C and *XRCC1* Arg194Trp wild type alleles.

#### High-risk groups

The high-risk multilocus genotypes were: IV: *OGG1* (IVS4-15C>):CG, *PTGS2* (-1195G>A):GA, MSH2 (-118T>C):CC and *OGG1* Ser326Cys:GG (onset 46-60 years); V: *OGG1* (IVS4-15C>):GG, and *OGG1* Ser326Cys:CG (onset 46–60 years); and VI: *ERCC2* Asp312Asn:AA, *ERCC2* Lys751Gln:CC and *TLR2* –196 to –174del:ID (onset 46–55 years) (Table S2).

*OGG1* (IVS4-15C>G) and *OGG1* Ser326Cys polymorphisms were the influential variants in defining group IV as indicated by elevated question relevance factor (QRF or the relevance of a variable to a pure type, with a lower limit of zero) scores of 1.26 and 1.21, respectively. This group was also homozygous for the *MSH2* promoter polymorphism at position -118 (CC). Group V were mostly females and had their age-of-onset as 46-60 years. They carried 100% probability for carrying *OGG1* (IVS4-15C>): GG genotype, and were 100% heterozygous for *OGG1* Ser326Cys polymorphism. The QRFs for these genetic variants were 1.66 and 1.21 respectively which defined the group. Group VI had age of onset at 46-55 years. There were more females (91%) in this group. All were homozygous for *ERCC2* Lys751Gln (CC) and *ERCC2* Asp312Asn (AA) with a QRF of 1.46 and 2.19, respectively. This group was also almost heterozygous for *TLR2* –196 to –174del (ID) polymorphism.

To find out the risk associated with each high-intrinsic set, we categorized the individuals on the basis of combination of genotypes that defined the set. Logistic regression analysis found that individuals in set I were at 3 fold increased risk of GBC (*P* = 0.033, 95% CI = 1.09-8.61). Individuals in set II were at 1.7 fold increased risk for GBC (*P* = 0.010, 95% CI = 1.13-2.51) while set III conferred >2 fold increased risk for GBC (*P* = 0.013, 95% CI = 1.18-4.16) (Table 5).

**Table 5:** Gallbladder cancer (GBC) risk associated with risk groups based on GoM.

Set	Genotype of Individuals in Each Set	OR^a^ (95% CI)	*P*-Value
**Set 1**	*OGG1*-326 (GG) + *OGG1*-IVS4-15C>G (CG) + *MSH2*-118T>C (CC) + *PTGS2*-1195G>A (GA)	3.07 (1.09–8.61)	0.033
**Set 2**	*OGG1*-326 (CG) + *OGG1*-IVS4-15C>G (GG)	1.69 (1.13–2.51)	0.010
**Set 3**	*ERCC2*-312 (AA) + *ERCC2*-751 (CC) + *TLR2*-Δ22 (ins,del)	2.22 (1.18–4.16)	0.013

a Adjusted for age and gender.

#### Informative variables

Information content for each variable was estimated by ‘H’ statistic (Shannon, Bell Laboratories). H close to 0 indicates similar outcome frequencies for each set (Table S2). Higher values represent increasing information content. *OGG1* (IVS4-15C>G), *OGG1* Ser326Cys, *ERCC2* Lys751Gln and *ERCC2* Asp312Asn had H >0.75. H>0.50 was seen in *MSH2* (IVS1+9G>C), *MSH2* (-118T>C) and *PTGS2* (-1195G>A). These are the variables strongly determining the risk for sets I to VI (Table S2).

#### Membership in the risk sets

Graded membership scores were automatically generated for each subject ranging from zero (no resemblance) to one (exact match) according to maximum likelihood estimation procedure. The sizes of the high intrinsic risk groups were 78.0, 55.9 and 36.5, respectively. The sizes of the low intrinsic risk groups were 80.5, 115.0 and 93.9, respectively (Table S2).

## Discussion

Carcinoma of the gallbladder (GBC) is a relatively rare malignancy with poor prognosis and high fatality rate affecting females two to three times more commonly than males.[2] Several epidemiologic studies have indicated the role of genetic factors in the pathogenesis of GBC by modifying the risk involved [24], [25], [26]. But most of these studies have provided conflicting results and there are difficulties in validating and replicating them.

GBC being a multifactorial and multistep disease, there may be a complex interaction between multiple risk alleles acting in a stronger manner in a combination rather than individually. So to achieve a more comprehensive evaluation of GBC risk considering several genetic variants simultaneously, present analysis was carried out in order to identify high and low intrinsic risk sets of gene variants. Of the included 16 polymorphisms, some of them were found to be significantly associated with GBC risk in our previous preliminary studies while others showed little or no influence on the risk for GBC development [4], [27], [28].

For the higher order gene-gene interaction analysis, we employed 2 statistical approaches namely CART and GoM analysis to find out the particular combinations of genetic variants contributing to GBC risk.

In CART analysis, which is a non-parametric statistical approach for conducting regression and classification analyses by recursive partitioning [29], the study subjects were grouped according to different risk levels on the basis of the different gene polymorphisms. From this analysis, we found that development of GBC involves complex genetic interactions among the DNA repair, apoptotic and inflammatory pathway gene variants. However, our results should be interpreted with caution because of the limited number of subjects in some of the CART terminal nodes.

In the GoM analysis, which includes all the predictors in a single model thus avoiding very large tested groups, and multiple comparison problems, six risk sets were identified for the present data defining three low intrinsic risk sets (I-III) and three high intrinsic risk sets (IV-VI), varying on the basis of age and significant risk alleles. The present approach automatically categorizes individuals to risk sets on the basis of graded membership scores.

The high intrinsic risk (sets IV to VI) was described by the presence of multiple risk alleles while the low intrinsic risk (sets I-III) was characterized by multiple protective alleles. Similarity to the high intrinsic risk sets carried ∼3-fold elevated risk for GBC.

Risk factors for Group IV were *OGG1* (IVS4-15C>):CG and *OGG1* Ser326Cys:GG. However, *PTGS2* (-1195G>A):GA and *MSH2* (-118T>C):CC were also relevant. Risk factors for Group V had *OGG1* (IVS4-15C>):CG and *OGG1* Ser326Cys:GG as the most prevalent risk factors of GBC. That for Group VI was the promoter polymorphism in *TLR2* at –196 to –174 (ID) along with *ERCC2* Asp312Asn:AA and *ERCC2* Lys751Gln:CC genotypes.

The GoM analysis revealed the importance of two *OGG1* polymorphisms along with *PTGS2* -1195G>A polymorphism in susceptibility to GBC risk. This was evident from the fact that the set IV and set V were harbouring both the *OGG1* polymorphisms.

The human DNA repair enzyme, OGG1, is a DNA glycosylase/AP lyase that efficiently repairs 8-hydroxy-2′-deoxyguanosine (8-OHdG), one of the most abundant oxidative products in DNA. 8-OHdG is highly mutagenic *in vitro* and *in vivo* giving rise to GC to TA transversions on DNA replication, which is frequent in several oncogenes and tumor suppressor genes [30], [31]. We had earlier reported an association between *OGG1* Ser326Cys polymorphism in a case-control study for GBC [28]. Also, significant association has been observed between *OGG1* Ser326Cys polymorphism and risk of esophageal [32], lung [33], bladder [34], breast [35] and colon [36] cancers. In addition to *OGG1* Ser326Cys, we also evaluated the influence of another polymorphism of *OGG1* gene present in Intron 4 (IVS4-15C>G). This is an intronic polymorphism residing in Intron 4 of *OGG1* gene and frequent allelic imbalance in this region has been shown to be involved in head and neck squamous carcinogenesis [37]. It seems that this polymorphism is involved in the splicing of *OGG1* mRNA. All the three high risk sets (IV, V and VI) were 100% carriers of the variant allele of *OGG1* polymorphisms.

PTGS2 over-expression has been observed in malignancies of various organs such as colorectum, lung, breast, prostate, bladder, stomach, and esophagus [38]. The *PTGS*2 promoter region contains binding sites for several key cis acting regulatory elements [39]. Present study signified the role of *PTGS2* -1195G>A polymorphism in GBC pathogenesis. Studies have shown that -1195A allele is able to bind c-MYB, one of the important transcription-factor that activates PTGS2 expression. c-MYB is required to keep a balance between cell division, differentiation and survival [40]. Luciferase assays have also shown significantly increased transcriptional activity of *PTGS2* gene in the individuals carrying -1195A allele [41], [42]. The elevated levels of PTGS2 leads to overproduction of prostaglandins (PGE_2_) which, being pro-carcinogenic, can prop up tumor growth by various signaling pathways controlling angiogenesis, cell proliferation, suppression of immune responses, invasiveness and also by inhibiting tumor cells apoptosis [43], [44], [45]. Another *PTGS2* polymorphism the -765G>C (rs20417), was also found to be modulating the risk of GBC in our multigenic model. This polymorphism is located within a putative *Stimulatory protein 1* (SP1) binding site that leads to a 30% reduction of the *PTGS2* promoter activity in vitro [46]. Although the exact molecular mechanism by which this polymorphism may affect the risk of GBC development is still unclear, studies in the *PTGS2* promoter revealed that *PTGS2* transcription is activated by E2 promoter binding factor 1 (E2F1) [47], which is dependent on the transactivation and DNA-binding domains of E2F1 [48]. So the ability of this polymorphism to create an E2F binding site, essential for the expression of several genes [49], might help us to understand why we observed increased risk.

The phenomenon that a combination of polymorphisms within genes of unrelated pathways may elevate the risk for GBC could be explained by two hypotheses. One possibility is that some connection between these genes or proteins exists but still remains to be discovered. Another hypothesis, more probable in our opinion, is that the genes influencing risk for GBC may as well comprise a set of alterations located within genes not related to each other. Such an unfavourable genetic profile could finally lead to appearance of the disease, although particular genes do not share any common functions and separately evoke a little or unnoticeable effect. Moreover, there may be multiple sufficient risk sets for GBC. Looking at multiple genes together rather than analyzing them individually may improve identification of risk alleles. In the present study, both GoM and CART categorized the GBC patients into high and low risk groups on the basis of analyzed polymorphisms.

Our study has several strengths like all our control subjects were under Hardy-Weinberg equilibrium minimizing population stratification, all our cases were histopathologically confirmed minimizing misclassification of outcome and cases and controls were matched for age and gender. The selection and survival biases in our study were negligible due to almost complete case ascertainment and high response rates. Strict quality control for genotyping was used, minimizing the potential for genotype misclassification. To limit potential confounding false positive associations due to moderate sample size and population stratification, we used software as described by Wacholder et al. [18] to filter out false-positive associations by setting very rigorous prior probabilities.

Limitations and sources of bias should be considered. Like all other case-control studies, inherent biases like selection bias and recall bias in the present study may have led to some spurious results. Although, the inclusion of SNPs in our study was based on potential functional role in genes with higher potential of being associated to cancer risk, a more comprehensive approach including tagging SNPs would present more convincing support for the associations. There is genetic variation within other genes we did not evaluate and the selected genes we studied in the study. However it is worth mentioning that the control cohort was gallstone negative which could present a bias in the obtained results as gallstone disease is an established risk factor for GBC. Also the relatively small sample size of our study might prevent some observed effects of genetic polymorphisms from reaching statistical significance. A more comprehensive approach including environmental factors would probably further improve the results.

In summary, the present study is one of the first to use a polygenic strategy to evaluate the involvement of DNA repair, apoptotic and inflammatory pathway gene polymorphisms in gallbladder carcinoma. Our results indicate that CART and GoM can play an important role as statistical tools in genetic association studies to detect unknown interactions among the risk-associated SNPs with marginal effects. Moreover, because the data is analyzed simultaneously, multiple comparisons are avoided. The advantage of using cluster analysis over CART relies on the fact that risk sets can be identified even with small sample size. Such studies of genetic interactions, along with their biological validations can be used to identify diagnostic procedures and early therapeutic interference so as to prevent or significantly delay the clinical manifestations of GBC. However, this study included only North Indian individuals, therefore the results may not apply to other ethnic groups.

To conclude, we identified high and low intrinsic risk profiles for GBC from information on multiple genetic variants modulating DNA repair, inflammation and apoptosis.

## Supporting Information

Table S1 **The genes and SNPs investigated.**
(DOC)

Table S2 **Gallbladder cancer (GBC) disease risk groups.**
(DOC)
