# Supplementary material for: Gallbladder Cancer Predisposition: A Multigenic Approach to DNA-Repair, Apoptotic and Inflammatory Pathway Genes
Source: PLoS One. 2011 Jan 21;6(1):e16449. doi: 10.1371/journal.pone.0016449 (PMC3025033; doi:10.1371/journal.pone.0016449)
Supplement: Table S1 — The genes and SNPs investigated. (DOC) [file pone.0016449.s001.doc]

Table S1. The genes and SNPs investigated.

| **Gene** | **Polymorphism** | **Forward primer (5′-3′)** | **Reverse primer (5′-3′)** | **Product** | **RE** | **Fragments** |
| --- | --- | --- | --- | --- | --- | --- |
| *ERCC2* | rs1799793 (G>A) | TCGGGGCTCACCCTGCAGCACTTCCT | CTGTTGGTGGGTGCCCGTATCTG TTGGTCT | 751 bp | *Sty*I | G= 507+ 474, A= 474+244+33 bp |
| *ERCC2* | rs13181 (A>C) | CTGCTCAGCCTGGAGCAGCTAGAATC AGAGGAGACGCTG | AAGACCTTCTAGCACCACCG | 161 bp | *Pst*I | A= 161, C= 120+41 bp |
| *MSH2* | rs2303426 (G>C) | GACCGGGGCGACTTCTATAC | AAAGGAGCCGCGCCACAAGG | 160 bp | *Bgl*I | G= 160, C= 113+47 bp |
| *MSH2* | rs2303425 (T>C) | GAAACGCAGCCCTGGAAGCTAA | AAACCTCCTCACCTCCTGGTTG | 136 bp | *Tsp509*I | T= 116+20, C= 136 bp |
| *OGG1* | rs1052133 (C>G) | ACTGTCACTAGTCTCACCAG | GGAAGGTGCTTGGGGAAT | 200 bp | *Fnu4H*I | C= 200, G= 100 bp |
| *OGG1* | rs2072668 (C>G) | CTTCTTCCACAAGGGCTGA | CACTGGGTCTGGGCTGGAGGAG | 292 bp | *Hinf*I | C= 292, G= 274+18 bp |
| *XRCC1* | rs1799782 (C>T) | GTTCCGTGTGAAGGAGGAGGA | CGAGTCTAGGTCTCAACCCTACTCACT | 138 bp | *Pvu*II | C= 138, T= 75+63 bp |
| *XRCC1* | rs25487 (A>G) | GCATCGTGCGTAAGGAGTG | CCTTCCCTCATCTGGAGTAC | 236 bp | *Msp*I | A=177+59, G= 236 bp |
| *CASP8* | rs3834129 (ins/del) | ACTCTGCATGCCAGGAGCTAAG | CCATAGTAATTCTTGCTCTGCCC | 179 bp | *Pvu*II | I= 179, D= 148+25 bp |
| *CASP8* | rs1045485 (G>C) | CATTTTGAGATCAAGCCCCGC | CCCTTGTCTCCATGGGAGAGGA | 132 bp | *BstU*I | G= 112 +20, C= 132 bp |
| *CASP8* | rs3769818 (G>A) | TCTGAGACAACCCATTTACC | AGAATATCATCGCCTCTGAA | 194 bp | *Ssp*I | A= 160+34, G= 194 bp |
| *PTGS2* | rs689466 (G>A) | GCCCTTCATAGGAGATACTGG | CCCTGAGCACTACCCATGAT | 272 bp | *Pvu*II | G= 272, A= 220+52 bp |
| *PTGS2* | rs20417 (G>C) | GCTAAGTTGCTTTCAACAGAAGAAAT | TATTATGACGAGAATTTACCTTTCGC | 100 bp | *Hha*I | G= 100, C= 75+25 bp |
| *PTGS2* | rs5275 (T>C) | GTTTGAAATTTTAAAGTACTTTTGAT | TTTCAAATTATTGTTTCATTGC | 147 bp | *Bcl*I | T= 147, C= 123+24 bp |
| *TLR2* | Δ22 (ins/del) | cacggaggcagcgagaaa | ctgggccgtgcaaagaag | 286 bp | - | I= 286, D= 264 bp |
| *TLR4* | rs4986791 (C>T) | GGTTGCTGTTCTCAAAGTGATTTTG GGAGAA | GGAAATCCAGATGTTCTAGTTGTTC TAAGCC | 124 bp | *Hinf*I | C= 124, T= 98 + 26 bp |

RE, Restriction enzyme

IS, Insertion specific primer
