# Supplementary material for: Gallbladder Cancer Predisposition: A Multigenic Approach to DNA-Repair, Apoptotic and Inflammatory Pathway Genes
Source: PLoS One. 2011 Jan 21;6(1):e16449. doi: 10.1371/journal.pone.0016449 (PMC3025033; doi:10.1371/journal.pone.0016449)
Supplement: Table S2 — Gallbladder cancer (GBC) disease risk groups. (DOC) [file pone.0016449.s002.doc]

Table S2. Gallbladder cancer (GBC) disease risk groups

| **Attributes** | **I** | **II** | **III** | **IV** | **V** | **VI** | **H** |
| --- | --- | --- | --- | --- | --- | --- | --- |
| **GBC** | 0 | 0 | 0 | 100 | 100 | 100 | 0.69 |
| **Age** | | | | | | | 0.17 |
| **<40** | 6 | 0 | 0 | 11 | 13 | 0 |  |
| **40-45** | 20 | 31 | 15 | 0 | 16 | 12 |  |
| **46-50** | 3 | 13 | 37 | 33 | 18 | 33 |  |
| **51-55** | 13 | 22 | 26 | 32 | 23 | 31 |  |
| **56-60** | 48 | 24 | 15 | 15 | 30 | 5 |  |
| **>60** | 10 | 10 | 7 | 9 | 0 | 19 |  |
| **Gender** | | | | | | | 0.06 |
| **Male** | 11 | 36 | 54 | 47 | 28 | 9 |  |
| **Female** | 89 | 64 | 46 | 53 | 72 | 91 |  |
| ***XRCC1* Arg399Gln** | | | | | | | 0.56 |
| **GG** | 42 | 0 | 0 | 100 | 0 | 0 |  |
| **GA** | 46 | 77 | 66 | 0 | 100 | 30 |  |
| **AA** | 12 | 23 | 34 | 0 | 0 | 70 |  |
| ***XRCC1* Arg194Trp** | | | | | | | 0.36 |
| **CC** | 100 | 100 | 100 | 92 | 100 | 0 |  |
| **CT** | 0 | 0 | 0 | 0 | 0 | 100 |  |
| **TT** | 0 | 0 | 0 | 8 | 0 | 0 |  |
| ***OGG1* (IVS4-15C>G)** | | | | | | | 0.91 |
| **CC** | 0 | 100 | 0 | 0 | 0 | 0 |  |
| **CG** | 100 | 0 | 100 | 100 | 0 | 0 |  |
| **GG** | 0 | 0 | 0 | 0 | 100 | 0 |  |
| ***OGG1* Ser326Cys** | | | | | | | 0.87 |
| **CC** | 0 | 100 | 100 | 0 | 0 | 0 |  |
| **CG** | 100 | 0 | 0 | 0 | 100 | 0 |  |
| **GG** | 0 | 0 | 0 | 100 | 0 | 100 |  |
| ***ERCC2* Asp312Asn** | | | | | | | 0.87 |
| **GG** | 0 | 0 | 100 | 100 | 0 | 0 |  |
| **GA** | 72 | 100 | 0 | 0 | 61 | 0 |  |
| **AA** | 28 | 0 | 0 | 0 | 39 | 100 |  |
| ***ERCC2* Lys751Gln** | | | | | | | 1.08 |
| **AA** | 0 | 0 | 100 | 0 | 0 | 0 |  |
| **AC** | 100 | 83 | 0 | 0 | 100 | 0 |  |
| **CC** | 0 | 17 | 0 | 100 | 0 | 100 |  |
| ***MSH2* (-118T>C)** | | | | | | | 0.58 |
| **TT** | 0 | 100 | 100 | 0 | 100 | 0 |  |
| **TC** | 86 | 0 | 0 | 0 | 0 | 100 |  |
| **CC** | 14 | 0 | 0 | 100 | 0 | 0 |  |
| ***MSH2* (IVS1+9G>C)** | | | | | | | 0.59 |
| **GG** | 32 | 59 | 0 | 36 | 0 | 0 |  |
| **GC** | 68 | 41 | 100 | 64 | 0 | 100 |  |
| **CC** | 0 | 0 | 0 | 0 | 100 | 0 |  |
| ***CASP8* -652 6N ins/del** | | | | | | | 0.32 |
| **II** | 53 | 56 | 15 | 100 | 100 | 50 |  |
| **ID** | 47 | 44 | 47 | 0 | 0 | 50 |  |
| **DD** | 0 | 0 | 38 | 0 | 0 | 0 |  |
| ***CASP8* (IVS12-19G>A)** | | | | | | | 0.04 |
| **GG** | 77 | 73 | 65 | 64 | 70 | 92 |  |
| **GA** | 23 | 21 | 35 | 36 | 30 | 8 |  |
| **AA** | 0 | 6 | 0 | 0 | 0 | 0 |  |
| ***CASP8* Asp302His** | | | | | | | 0.28 |
| **GG** | 100 | 100 | 100 | 100 | 97 | 0 |  |
| **GC** | 0 | 0 | 0 | 0 | 0 | 100 |  |
| **CC** | 0 | 0 | 0 | 0 | 3 | 0 |  |
| ***TLR4* Thr399Ile** | | | | | | | 0.29 |
| **CC** | 100 | 100 | 100 | 100 | 97 | 0 |  |
| **CT** | 0 | 0 | 0 | 0 | 0 | 96 |  |
| **TT** | 0 | 0 | 0 | 0 | 3 | 4 |  |
| ***TLR2* –196 to –174del** | | | | | | | 0.65 |
| **II** | 100 | 100 | 0 | 100 | 83 | 0 |  |
| **ID** | 0 | 0 | 100 | 0 | 0 | 98 |  |
| **DD** | 0 | 0 | 0 | 0 | 17 | 2 |  |
| ***PTGS2* (Ex10+837T>C)** | | | | | | | 0.28 |
| **TT** | 36 | 43 | 37 | 0 | 27 | 100 |  |
| **TC** | 64 | 34 | 41 | 100 | 30 | 0 |  |
| **CC** | 0 | 23 | 22 | 0 | 43 | 0 |  |
| ***PTGS2* (-1195G>A)** | | | | | | | 0.52 |
| **GG** | 100 | 100 | 100 | 0 | 30 | 100 |  |
| **GA** | 0 | 0 | 0 | 85 | 70 | 0 |  |
| **AA** | 0 | 0 | 0 | 15 | 0 | 0 |  |
| ***PTGS2* (-765G>C)** | | | | | | | 0.29 |
| **GG** | 100 | 100 | 87 | 35 | 73 | 53 |  |
| **GC** | 0 | 0 | 13 | 65 | 27 | 0 |  |
| **CC** | 0 | 0 | 0 | 0 | 0 | 47 |  |
| **Group sizes** | 80.5 | 115.0 | 93.9 | 78.0 | 55.9 | 36.5 |  |

The risk sets are defined by the displayed probabilities.

H denotes information content for the variable, e.g., *OGG1* (IVS4-15C>) had the highest information content among the genetic variables; whereas *CASP8* (Ex13+51G>C) was the least informative (H =0.09); zero denotes no information.

Group size is the sum of membership in the group over all the subjects.
